# Supplementary material for: Recent Advances in Urinary Peptide and Proteomic Biomarkers in Chronic Kidney Disease: A Systematic Review
Source: Int J Mol Sci. 2023 May 23;24(11):9156. doi: 10.3390/ijms24119156 (PMC10252389; doi:10.3390/ijms24119156)
Supplement: Supplementary file 1 [file ijms-24-09156-s001.zip › ijms-2371390-supplementary.pdf]

Key	Item Type	Publication Year
HMMK9SMI	journalArticle	2018
KWYXLFLW	journalArticle	2021
E8MYKIF9	journalArticle	2018
MDRHTC5D	journalArticle	2017
9UL7TBV6	journalArticle	2017
7TUQQZBE	journalArticle	2018
RQSNHF6E	journalArticle	2019
LH5PV353	journalArticle	2019
W4BFBU9D	journalArticle	2017
BC8SYZKK	journalArticle	2018
YUYS8XCU	journalArticle	2020
SBKVZM8S	journalArticle	2019
7RYB3DZN	journalArticle	2022
S4EVK9AV	journalArticle	2022
88MWPHMS	journalArticle	2017
3K29YZYA	journalArticle	2018
C3J3VF3N	journalArticle	2021
2QMB2UEV	journalArticle	2020
7FFYBR3Y	journalArticle	2021
IWC2ZY3J	journalArticle	2021
G6BPI5X7	journalArticle	2021
AZF8N9YT	journalArticle	2017
QCX5JDI2	journalArticle	2018
WH7ZBV6A	journalArticle	2019
EECXSSG8	journalArticle	2021
88W4XYXV	journalArticle	2021
UILNQIKU	journalArticle	2021
MA88DR2K	journalArticle	2021
KE3IFX9I	journalArticle	2021
W3F3J2G6	journalArticle	2021
MFCZDXQM	journalArticle	2021
CK94Z567	journalArticle	2021
UZJU3FD7	journalArticle	2021
NI7VW6CC	journalArticle	2020
QVA7AAF2	journalArticle	2020
J4UCBNG4	journalArticle	2020
2ZC5WI56	journalArticle	2020
869AJ2AC	journalArticle	2020
55BZBMY9	journalArticle	2020
J76FG9C6	journalArticle	2019
A5R2YBF7	journalArticle	2019
KWIQGR53	journalArticle	2019
KFFBSC6Z	journalArticle	2019
NMMPP5Y4	journalArticle	2018
4UT8EJZ3	journalArticle	2018
GWF9FBMF	journalArticle	2018
SARUB7S3	journalArticle	2018
DW45EQSR	journalArticle	2018
SPUXAECV	journalArticle	2018

IERNU4DT	journalArticle	2018
C6VHAUNW	journalArticle	2017
CWRINXYM	journalArticle	2017
D9YNEBAI	journalArticle	2017
HH6ANWU2	journalArticle	2021
DIWJ9ITM	journalArticle	2017
4XA8XFZN	journalArticle	2017
CCZK9VC3	journalArticle	2022
2D4PWNDN	journalArticle	2016
PQLFTUY9	journalArticle	2022
G23KNTGG	journalArticle	2008
WIX3FDU6	journalArticle	2019
	journalArticle	2018

Author
Pejchinovski, M.; Siwy, J.; Mullen, W.; Mischak, H.; Petri, M. A.; Burkly, L. C.; Wei, R.
Mavrogeorgis, Emmanouil; Mischak, Harald; Latosinska, Agnieszka; Vlahou, Antonia; Schanstra, Joost F
Magalhães, Pedro; Pontillo, Claudia; Pejchinovski, Martin; Siwy, Justyna; Krochmal, Magdalena; Makrid
Magalhães, Pedro; Pejchinovski, Martin; Markoska, Katerina; Banasik, Miroslaw; Klinger, Marian; Švec-
Lindhardt, Morten; Persson, Frederik; Zürbig, Petra; Stalmach, Angelique; Mischak, Harald; de Zeeuw,
Currie, Gemma E.; von Scholten, Bernt Johan; Mary, Sheon; Flores Guerrero, Jose-Luis; Lindhardt, Mor
Brunner, Hermine I.; Gulati, Gaurav; Klein-Gitelman, Marisa S.; Rouster-Stevens, Kelly A.; Tucker, Lori;
Takei, Yoshinori; Takahashi, Shunsuke; Nakasatomi, Masao; Sakairi, Toru; Ikeuchi, Hidekazu; Kaneko, Y
Pérez, Vanessa; López, Dolores; Boixadera, Ester; Ibernón, Meritxell; Espinal, Anna; Bonet, Josep; Rom
Ling, Chen; Liu, Xiaorong; Shen, Ying; Chen, Zhi; Fan, Jianfeng; Jiang, Yeping; Meng, Qun
Gonzalez Guerrico, Anatilde M.; Lieske, John; Klee, George; Kumar, Sanjay; Lopez-Baez, Victor; Wright,
Yang, Joshua Y. C.; Sarwal, Reuben D.; Fervenza, Fernando C.; Sarwal, Minnie M.; Lafayette, Richard A.
Melchinger, Hannah; Calderon-Gutierrez, Frida; Obeid, Wassim; Xu, Leyuan; Shaw, Melissa M.; Lucianc
Ou, Shuo-Ming; Tsai, Ming-Tsun; Chen, Huan-Yuan; Li, Fu-An; Lee, Kuo-Hua; Tseng, Wei-Cheng; Chang,
Zhou, Dong; Tian, Yuan; Sun, Ling; Zhou, Lili; Xiao, Liangxiang; Tan, Roderick J.; Tian, Jianwei; Fu, Haiyar
Rodríguez-Ortiz, María E.; Pontillo, Claudia; Rodríguez, Mariano; Zürbig, Petra; Mischak, Harald; Ortiz, .
Sánchez-Álamo, Beatriz; García-Iñigo, Francisco José; Shabaka, Amir; Acedo, Juan Manuel; Cases-Coror
Tofte, Nete; Lindhardt, Morten; Adamova, Katarina; Bakker, Stephan J. L.; Beige, Joachim; Beulens, Jol
Catanese, Lorenzo; Siwy, Justyna; Mavrogeorgis, Emmanouil; Amann, Kerstin; Mischak, Harald; Beige,
Wendt, Ralph; Siwy, Justyna; He, Tianlin; Latosinska, Agnieszka; Wiech, Thorsten; Zipfel, Peter F.; Tserg
Rudnicki, Michael; Siwy, Justyna; Wendt, Ralph; Lipphardt, Mark; Koziolek, Michael J.; Maixnerova, Dit
Pejchinovski, Martin; Siwy, Justyna; Metzger, Jochen; Dakna, Mohammed; Mischak, Harald; Klein, Julie
Lindhardt, Morten; Persson, Frederik; Oxlund, Christina; Jacobsen, Ib A.; Zürbig, Petra; Mischak, Harald
Zürbig, Petra; Mischak, Harald; Menne, Jan; Haller, Hermann
Puthumana, Jeremy; Thiessen-Philbrook, Heather; Xu, Leyuan; Coca, Steven G.; Garg, Amit X.; Himmel
Wörn, Matthias; Bohnert, Bernhard N.; Alenazi, Fawza; Boldt, Karsten; Klose, Franziska; Junger, Katrin;
Menez, Steven; Ju, Wenjun; Menon, Rajasree; Moledina, Dennis G.; Thiessen Philbrook, Heather; McA
Davies, Jennifer C.; Carlsson, Emil; Midgley, Angela; Smith, Eve M. D.; Bruce, Ian N.; Beresford, Michae
Perez-Gomez, Maria Vanessa; Pizarro-Sanchez, Soledad; Gracia-Iguacel, Carolina; Cano, Santiago; Canr
Norvik, Jon Viljar; Harskamp, Laura R.; Nair, Viji; Shedden, Kerby; Solbu, Marit D.; Eriksen, Bjørn O.; Kre
Seibert, Felix S.; Heringhaus, Anja; Pagonas, Nikolaos; Rohn, Benjamin; Bauer, Frederic; Trappe, Hans-J
Mejia-Vilet, Juan M.; Shapiro, John P.; Zhang, Xiaolan L.; Cruz, Cristino; Zimmerman, Grant; Méndez-Pé
Lee, Yu Ho; Kim, Ki Pyo; Park, Sun-Hwa; Kim, Dong-Jin; Kim, Yang-Gyun; Moon, Ju-Young; Jung, Su-Woo
Wu, Liang; Li, Xiao-Qian; Chang, Dong-Yuan; Zhang, Huifen; Li, Jun-Juan; Wu, Shou-Ling; Zhang, Lu-Xia;
Brondani, Letícia de Almeida; Soares, Ariana Aguiar; Recamonde-Mendoza, Mariana; Dall'Agnol, Angél
Ahn, Hee-Sung; Kim, Jong Ho; Jeong, Hwangkyo; Yu, Jiyoung; Yeom, Jeonghun; Song, Sang Heon; Kim,
Doykov, Ivan D.; Heywood, Wendy E.; Nikolaenko, Valeria; Śpiewak, Justyna; Hällqvist, Jenny; Clayton,
Gohda, Tomohito; Kamei, Nozomu; Koshida, Takeo; Kubota, Mitsunobu; Tanaka, Kanako; Yamashita, Y
Duan, Suyan; Chen, Jiajia; Wu, Lin; Nie, Guangyan; Sun, Lianqin; Zhang, Chengning; Huang, Zhimin; Xin
Schunk, Stefan J.; Zarbock, Alexander; Meersch, Melanie; Küllmar, Mira; Kellum, John A.; Schmit, Davic
Li, Aimei; Yi, Bin; Liu, Yan; Wang, Jianwen; Dai, Qing; Huang, Yuxi; Li, Yan Chun; Zhang, Hao
Satirapoj, Bancha; Pooluea, Pimanong; Nata, Naowanit; Supasyndh, Ouppatham
Pilemann-Lyberg, Sascha; Rasmussen, Daniel Guldager Kring; Hansen, Tine Willum; Tofte, Nete; Winth
Zewinger, Stephen; Rauen, Thomas; Rudnicki, Michael; Federico, Giuseppina; Wagner, Martina; Triem
Żyłka, Agnieszka; Dumnicka, Paulina; Kuśnierz-Cabala, Beata; Gala-Błądzińska, Agnieszka; Ceranowicz,
Satirapoj, Bancha; Dispan, Rattanawan; Radinahamed, Piyanuch; Kitiyakara, Chagriya
Liao, Wen-Ling; Chang, Chiz-Tzung; Chen, Ching-Chu; Lee, Wen-Jane; Lin, Shih-Yi; Liao, Hsin-Yi; Wu, Ch
Li, Baihong; Zhang, Yanqin; Wang, Fang; Nair, Viji; Ding, Fangrui; Xiao, Huijie; Yao, Yong; Kretzler, Matt
Critselis, Elena; Vlahou, Antonia; Stel, Vianda S.; Morton, Rachael L.

Caterino, Marianna; Zacchia, Miriam; Costanzo, Michele; Bruno, Giuliana; Arcaniolo, Davide; Trepiccio
Yang, Xiaobing; Chen, Chunbo; Teng, Siyuan; Fu, Xiaorui; Zha, Yan; Liu, Huafeng; Wang, Li; Tian, Jianwe
Dong, Liqun; Ma, Qing; Bennett, Michael; Devarajan, Prasad
Rasmussen, Daniel Guldager Kring; Fenton, Anthony; Jesky, Mark; Ferro, Charles; Boor, Peter; Tepel, M
Verbeke, Francis; Siwy, Justyna; Van Biesen, Wim; Mischak, Harald; Pletinck, Anneleen; Schepers, Eva;
Siwy, Justyna; Zürbig, Petra; Argiles, Angel; Beige, Joachim; Haubitz, Marion; Jankowski, Joachim; Julia
Pontillo, Claudia; Zhang, Zhen-Yu; Schanstra, Joost P.; Jacobs, Lotte; Zürbig, Petra; Thijs, Lutgarde; Ram
Petra, Eleni; Siwy, Justyna; Vlahou, Antonia; Jankowski, Joachim
Federico, Giuseppina; Meister, Michael; Mathow, Daniel; Heine, Gunnar H.; Moldenhauer, Gerhard; Po
Cortvrindt, Charlotte; Speeckaert, Reinhart; Delanghe, Joris R.; Speeckaert, Marijn M.
Bolignano, Davide; Donato, Valentina; Coppolino, Giuseppe; Campo, Susanna; Buemi, Antoine; Lacqua
Watson, Drew; Yang, Joshua Y. C.; Sarwal, Reuben D.; Sigdel, Tara K.; Liberto, Juliane M.; Damm, Izabe
Nowak N, Skupien J, Smiles AM, Yamanouchi M, Niewczas MA, Galecki AT, Duffin KL, Breyer MD, Pulle

Title
Urine peptidomic biomarkers for diagnosis of patients with systematic lupus erythematosus
Collagen-Derived Peptides in CKD: A Link to Fibrosis
Comparison of Urine and Plasma Peptidome Indicates Selectivity in Renal Peptide Handling
Association of kidney fibrosis with urinary peptides: a path towards non-invasive liquid biopsies?
Urinary proteomics predict onset of microalbuminuria in normoalbuminuric type 2 diabetic patients, a
Urinary proteomics for prediction of mortality in patients with type 2 diabetes and microalbuminuria
Urine biomarkers of chronic kidney damage and renal functional decline in childhood-onset systemic l
Urinary Activin A is a novel biomarker reflecting renal inflammation and tubular damage in ANCA-asso
Comparative differential proteomic analysis of minimal change disease and focal segmental glomerulo
Urinary CD80 excretion is a predictor of good outcome in children with primary nephrotic syndrome
Urinary CD80 Discriminates Among Glomerular Disease Types and Reflects Disease Activity
Noninvasive Urinary Monitoring of Progression in IgA Nephropathy
Urine Uromodulin as a Biomarker of Kidney Tubulointerstitial Fibrosis
Urinary Galectin-3 as a Novel Biomarker for the Prediction of Renal Fibrosis and Kidney Disease Progre
Matrix Metalloproteinase-7 Is a Urinary Biomarker and Pathogenic Mediator of Kidney Fibrosis
Novel Urinary Biomarkers For Improved Prediction Of Progressive Egfr Loss In Early Chronic Kidney Dis
Urinary Dickkopf-3: a new biomarker for CKD progression and mortality
Early detection of diabetic kidney disease by urinary proteomics and subsequent intervention with spi
A Novel Urinary Proteomics Classifier for Non-Invasive Evaluation of Interstitial Fibrosis and Tubular At
Molecular Mapping of Urinary Complement Peptides in Kidney Diseases
Urine proteomics for prediction of disease progression in patients with IgA nephropathy
Urine peptidome analysis predicts risk of end-stage renal disease and reveals proteolytic pathways inv
Predicting albuminuria response to spironolactone treatment with urinary proteomics in patients with
CKD273 Enables Efficient Prediction of Diabetic Nephropathy in Nonalbuminuric Patients
Biomarkers of inflammation and repair in kidney disease progression
Proteasuria in nephrotic syndrome-quantification and proteomic profiling
Urinary EGF and MCP-1 and risk of CKD after cardiac surgery
A panel of urinary proteins predicts active lupus nephritis and response to rituximab treatment
Urinary Growth Differentiation Factor-15 (GDF15) levels as a biomarker of adverse outcomes and biop
Urinary excretion of epidermal growth factor and rapid loss of kidney function
Dickkopf-3 in the prediction of contrast media induced acute kidney injury
Association Between Urinary Epidermal Growth Factor and Renal Prognosis in Lupus Nephritis
Urinary chemokine C-X-C motif ligand 16 and endostatin as predictors of tubulointerstitial fibrosis in p
Associations of urinary epidermal growth factor and monocyte chemotactic protein-1 with kidney invo
Urinary peptidomics and bioinformatics for the detection of diabetic kidney disease
Differential Urinary Proteome Analysis for Predicting Prognosis in Type 2 Diabetes Patients with and w
Rapid, proteomic urine assay for monitoring progressive organ disease in Fabry disease
Circulating kidney injury molecule-1 as a biomarker of renal parameters in diabetic kidney disease
Assessment of urinary NGAL for differential diagnosis and progression of diabetic kidney disease
Association between urinary dickkopf-3, acute kidney injury, and subsequent loss of kidney function ir
Urinary NGAL and RBP Are Biomarkers of Normoalbuminuric Renal Insufficiency in Type 2 Diabetes Me
Urinary biomarkers of tubular injury to predict renal progression and end stage renal disease in type 2
Markers of Collagen Formation and Degradation Reflect Renal Function and Predict Adverse Outcome
Dickkopf-3 (DKK3) in Urine Identifies Patients with Short-Term Risk of eGFR Loss
Markers of Glomerular and Tubular Damage in the Early Stage of Kidney Disease in Type 2 Diabetic Pat
Urinary epidermal growth factor, monocyte chemoattractant protein-1 or their ratio as predictors for
Urinary Proteomics for the Early Diagnosis of Diabetic Nephropathy in Taiwanese Patients
Urinary epidermal growth factor as a prognostic marker for the progression of Alport syndrome in chil
Cost-effectiveness of screening type 2 diabetes patients for chronic kidney disease progression with th

Urine Proteomics Revealed a Significant Correlation Between Urine-Fibronectin Abundance and Estima
Urinary Matrix Metalloproteinase-7 Predicts Severe AKI and Poor Outcomes after Cardiac Surgery
Urinary biomarkers of cell cycle arrest are delayed predictors of acute kidney injury after pediatric card
Urinary endotrophin predicts disease progression in patients with chronic kidney disease
The urinary proteomics classifier chronic kidney disease 273 predicts cardiovascular outcome in patier
Noninvasive diagnosis of chronic kidney diseases using urinary proteome analysis
Prediction of Chronic Kidney Disease Stage 3 by CKD273, a Urinary Proteomic Biomarker
Urine peptidome in combination with transcriptomics analysis highlights MMP7, MMP14 and PCSK5 fo
Tubular Dickkopf-3 promotes the development of renal atrophy and fibrosis
Urinary Epidermal Growth Factor: A Promising “Next Generation” Biomarker in Kidney Disease
Neutrophil Gelatinase–Associated Lipocalin (NGAL) as a Marker of Kidney Damage
A Novel Multi-Biomarker Assay for Non-Invasive Quantitative Monitoring of Kidney Injury
Markers of early progressive renal decline in type 2 diabetes suggest different implications for etiologi

Publication Title
Lupus
Toxins
Proteomics. Clinical Applications
Scientific Reports
Nephrology, Dialysis, Transplantation: Official Publication of the European Dialysis and Transplant Asso
Cardiovascular Diabetology
Pediatric Nephrology (Berlin, Germany)
PloS One
BMC nephrology
Pediatric Nephrology (Berlin, Germany)
Kidney International Reports
International Journal of Molecular Sciences
Clinical journal of the American Society of Nephrology: CJASN
Biomedicines
Journal of the American Society of Nephrology: JASN
Scientific Reports
Nephrology, Dialysis, Transplantation: Official Publication of the European Dialysis and Transplant Asso
The Lancet. Diabetes & Endocrinology
Proteomes
Proteomes
Nephrology, Dialysis, Transplantation: Official Publication of the European Dialysis and Transplant Asso
Nephrology, Dialysis, Transplantation: Official Publication of the European Dialysis and Transplant Asso
Nephrology, Dialysis, Transplantation: Official Publication of the European Dialysis and Transplant Asso
Diabetes Care
The Journal of Clinical Investigation
Journal of Proteomics
JCI insight
Rheumatology (Oxford, England)
Journal of Nephrology
Nephrology, Dialysis, Transplantation: Official Publication of the European Dialysis and Transplant Asso
Journal of Nephrology
Arthritis & Rheumatology (Hoboken, N.J.)
Nephrology, Dialysis, Transplantation: Official Publication of the European Dialysis and Transplant Asso
Nephrology, Dialysis, Transplantation: Official Publication of the European Dialysis and Transplant Asso
Scientific Reports
International Journal of Molecular Sciences
Journal of Medical Genetics
Journal of Diabetes Investigation
Journal of Diabetes and Its Complications
Lancet (London, England)
Journal of Immunology Research
Journal of Diabetes and Its Complications
Diabetes Care
Journal of the American Society of Nephrology: JASN
Mediators of Inflammation
BMC nephrology
Journal of Clinical Medicine
Pediatric Nephrology (Berlin, Germany)
Nephrology, Dialysis, Transplantation: Official Publication of the European Dialysis and Transplant Asso

Kidney & Blood Pressure Research
Journal of the American Society of Nephrology: JASN
Pediatric Nephrology (Berlin, Germany)
Scientific Reports
Nephrology, Dialysis, Transplantation: Official Publication of the European Dialysis and Transplant Asso
Nephrology, Dialysis, Transplantation: Official Publication of the European Dialysis and Transplant Asso
Kidney International Reports
PloS One
JCI insight
American Journal of Nephrology
American Journal of Kidney Diseases
Journal of Clinical Medicine
Kidney International

ISBN	ISSN	DOI
	1477-0962	10.1177/0961203317707827
	2072-6651	10.3390/toxins14010010
	1862-8354	10.1002/prca.201700163
	2045-2322	10.1038/s41598-017-17083-w
	1460-2385	10.1093/ndt/gfw292
	1475-2840	10.1186/s12933-018-0697-9
	1432-198X	10.1007/s00467-018-4049-5
	1932-6203	10.1371/journal.pone.0223703
	1471-2369	10.1186/s12882-017-0452-6
	1432-198X	10.1007/s00467-018-3885-7
	2468-0249	10.1016/j.ekir.2020.08.001
	1422-0067	10.3390/ijms20184463
	1555-905X	10.2215/CJN.04360422
	2227-9059	10.3390/biomedicines10030585
	1533-3450	10.1681/ASN.2016030354
	2045-2322	10.1038/s41598-018-34386-8
	1460-2385	10.1093/ndt/gfab198
	2213-8595	10.1016/S2213-8587(20)30026-7
	2227-7382	10.3390/proteomes9030032
	2227-7382	10.3390/proteomes9040049
	1460-2385	10.1093/ndt/gfaa307
	1460-2385	10.1093/ndt/gfw243
	1460-2385	10.1093/ndt/gfw406
	1935-5548	10.2337/dc18-1322
	1558-8238	10.1172/JCI139927
	1876-7737	10.1016/j.jprot.2020.103981
	2379-3708	10.1172/jci.insight.147464
	1462-0332	10.1093/rheumatology/keaa851
	1724-6059	10.1007/s40620-021-01020-2
	1460-2385	10.1093/ndt/gfaa208
	1724-6059	10.1007/s40620-020-00910-1
	2326-5205	10.1002/art.41507
	1460-2385	10.1093/ndt/gfz168
	1460-2385	10.1093/ndt/gfy314
	2045-2322	10.1038/s41598-020-58067-7
	1422-0067	10.3390/ijms21124236
	1468-6244	10.1136/jmedgenet-2019-106030
	2040-1124	10.1111/jdi.13139
	1873-460X	10.1016/j.jdiacomp.2020.107665
	1474-547X	10.1016/S0140-6736(19)30769-X
	2314-7156	10.1155/2019/5063089
	1873-460X	10.1016/j.jdiacomp.2019.05.013
	1935-5548	10.2337/dc18-2599
	1533-3450	10.1681/ASN.2018040405
	1466-1861	10.1155/2018/7659243
	1471-2369	10.1186/s12882-018-1043-x
	2077-0383	10.3390/jcm7120483
	1432-198X	10.1007/s00467-018-3988-1
	1460-2385	10.1093/ndt/gfx068

	1423-0143	10.1159/000488096
	1533-3450	10.1681/ASN.2017020142
	1432-198X	10.1007/s00467-017-3748-7
	2045-2322	10.1038/s41598-017-17470-3
	1460-2385	10.1093/ndt/gfz242
	1460-2385	10.1093/ndt/gfw337
	2468-0249	10.1016/j.ekir.2017.06.004
	1932-6203	10.1371/journal.pone.0262667
	2379-3708	10.1172/jci.insight.84916
	0250-8095, 1421-9670	10.1159/000524586
	02726386	10.1053/j.ajkd.2008.01.020
	2077-0383	10.3390/jcm8040499
		10.1016/j.kint.2017.11.024.

Url

https://www.karger.com/Article/FullText/524586
https://linkinghub.elsevier.com/retrieve/pii/S0272638608001637
https://www.mdpi.com/2077-0383/8/4/499

Abstract Note
Background Systematic lupus erythematosus (SLE) is characterized with various complications which c
Collagen is a major component of the extracellular matrix (ECM) and has an imminent role in fibrosis, i
PURPOSE: Urine is considered to be produced predominantly as a result of plasma filtration in the kidn
Chronic kidney disease (CKD) is a prevalent cause of morbidity and mortality worldwide. A hallmark of
BACKGROUND: Early prevention of diabetic nephropathy is not successful as early interventions have s
BACKGROUND: The urinary proteomic classifier CKD273 has shown promise for prediction of progress
OBJECTIVES: To delineate urine biomarkers that reflect kidney structural damage and predict renal fun
Activin A, a member of the transforming growth factor-beta superfamily, is a critical modulator of infla
BACKGROUND: Minimal change disease (MCD) and primary focal segmental glomerulosclerosis (FSGS)
BACKGROUND: The level of urinary cluster of differentiation 80 (uCD80) is elevated in most children w
INTRODUCTION: Heterogeneity of nephrotic diseases and a lack of validated biomarkers limits interven
Standard methods for detecting and monitoring of IgA nephropathy (IgAN) have conventionally requir
BACKGROUND AND OBJECTIVES: Uromodulin, produced exclusively in the kidney's thick ascending lim
Plasma galectin-3 (Gal-3) is associated with organ fibrosis, but whether urinary Gal-3 is a potential bior
Matrix metalloproteinase-7 (MMP-7), a secreted zinc- and calcium-dependent endopeptidase, is a tran
Chronic kidney disease is associated with increased risk of CKD progression and death. Therapeutic ap
BACKGROUND: Kidney fibrosis has been reported to be a prognostic factor in chronic kidney disease (
BACKGROUND: Microalbuminuria is an early sign of kidney disease in people with diabetes and indicat
Non-invasive urinary peptide biomarkers are able to detect and predict chronic kidney disease (CKD). I
Defective complement activation has been associated with various types of kidney disease. This led to
BACKGROUND: Risk of kidney function decline in immunoglobulin A (IgA) nephropathy (IgAN) is signifi
BACKGROUND: Autosomal dominant polycystic kidney disease (ADPKD) is characterized by slowly prog
BACKGROUND: The mineralocorticoid receptor antagonist spironolactone significantly reduces albumi
INTRODUCTIONAcute kidney injury and chronic kidney disease (CKD) are common in hospitalized patie
Nephrotic syndrome is characterized by urinary excretion of plasma proteases or proteasuria. There is
BACKGROUNDAssessment of chronic kidney disease (CKD) risk after acute kidney injury (AKI) is based
OBJECTIVES: 30% of patients with SLE develop LN. Presence and/or severity of LN are currently asses
BACKGROUND: Growth Differentiation Factor-15 (GDF15) is a member of the TGF-β superfamily. Incre
BACKGROUND: Lower urinary excretion of the kidney tubule-specific biomarker epidermal growth fact
BACKGROUND: Dickkopf-3 (DKK3) has recently been discovered as a urinary biomarker for the predict
OBJECTIVE: To evaluate the role of urinary epidermal growth factor (EGF) as a biomarker of chronic kic
BACKGROUND: Interstitial fibrosis and tubular atrophy (IFTA) is a well-recognized risk factor for poor r
BACKGROUND: In diabetic kidney disease (DKD), it is important to find biomarkers for predicting initiat
The aim of this study was to establish a peptidomic profile based on LC-MS/MS and random forest (RF
Renal dysfunction, a major complication of type 2 diabetes, can be predicted from estimated glomerul
BACKGROUND: Fabry disease is a progressive multisystemic disease, which affects the kidney and card
AIMS/INTRODUCTION: Urinary kidney injury molecule-1 (KIM-1) has been associated with proximal tul
OBJECTIVE: Chronic kidney disease (CKD) related to diabetes has become more common than glomeru
BACKGROUND: Cardiac surgery is associated with a high risk of postoperative acute kidney injury (AKI)
OBJECTIVES: As a screening index of diabetic kidney disease (DKD), urinary albumin/creatine ratio (UA
BACKGROUND: Novel potential tubular biomarkers in diabetic nephropathy could improve risk stratific
OBJECTIVE: Patients with type 1 diabetes (T1D) have a higher risk of developing chronic kidney disease
BACKGROUND: The individual course of CKD may vary, and improved methods for identifying which pa
Diabetic kidney disease develops in half of genetically predisposed patients with type 2 diabetes (T2DI
BACKGROUND: Increased monocyte chemoattractant protein-1 (MCP-1) and decreased epidermal gro
Diabetic nephropathy (DN) is a major complication in diabetic patients. Microalbuminuria testing is us
BACKGROUND: Alport syndrome is a rare hereditary kidney disease manifested with progressive renal
BACKGROUND: In type 2 diabetes mellitus (T2DM) patients, chronic kidney disease (CKD) progression

BACKGROUND: /Aims: Renal disease is a common cause of morbidity in patients with Bardet-Biedl syn
Urinary matrix metalloproteinase-7 (uMMP-7) levels consistently reflect the activity of intrarenal Wnt/
BACKGROUND: Several novel biomarkers that predict acute kidney injury (AKI) have recently been pro
Renal fibrosis is the central pathogenic process in progression of chronic kidney disease (CKD). Collage
BACKGROUND: The urinary proteomic classifier chronic kidney disease 273 (CKD273) is predictive for t
BACKGROUND: In spite of its invasive nature and risks, kidney biopsy is currently required for precise d
INTRODUCTION: CKD273 is a urinary biomarker, which in advanced chronic kidney disease predicts fur
Chronic kidney disease (CKD) is characterized by the loss of kidney function. The molecular mechanism
Renal tubular atrophy and interstitial fibrosis are common hallmarks of etiologically different progress
**<i>Background:</i>** The epidermal growth factor (EGF) is a globular protein that is generated
The current standard of care measures for kidney function, proteinuria, and serum creatinine (SCr) are

Date	Date Added	Date Modified	Access Date	Pages	Num Pages
2018-01	2023-01-18 21:31	2023-01-18 21:31		6-16	
2021-12-23	2023-01-18 21:31	2023-01-18 21:31		10	
2018-09	2023-01-18 21:31	2023-01-18 21:31		e1700163	
2017-12-05	2023-01-18 21:31	2023-01-18 21:31		16915	
2017-11-01	2023-01-18 21:30	2023-01-18 21:30		1866-1873	
2018-04-06	2023-01-18 21:30	2023-01-18 21:30		50	
2019-01	2023-01-19 09:43	2023-01-19 09:43		117-128	
2019	2023-01-19 09:41	2023-01-19 09:41		e0223703	
2017-02-03	2023-01-19 09:29	2023-01-19 09:29		49	
2018-07	2023-01-19 09:29	2023-01-19 09:29		1183-1187	
2020-11	2023-01-19 09:28	2023-01-19 09:28		2021-2031	
2019-09-10	2023-01-19 09:24	2023-01-19 09:24		4463	
2022-09	2023-01-19 09:21	2023-01-19 09:21		1284-1292	
2022-03-02	2023-01-19 09:20	2023-01-19 09:20		585	
2017-02	2023-01-19 09:19	2023-01-19 09:19		598-611	
2018-10-29	2023-01-19 09:15	2023-01-19 09:15		15940	
2021-12-02	2023-01-19 09:13	2023-01-19 09:13		2199-2207	
2020-04	2023-01-19 08:54	2023-01-19 08:54		301-312	
2021-07-13	2023-01-19 08:53	2023-01-19 08:53		32	
2021-12-13	2023-01-19 08:53	2023-01-19 08:53		49	
2021-12-31	2023-01-19 08:53	2023-01-19 08:53		42-52	
2017-03-01	2023-01-19 08:52	2023-01-19 08:52		487-497	
2018-02-01	2023-01-19 08:52	2023-01-19 08:52		296-303	
2019-01	2023-01-19 08:52	2023-01-19 08:52		e4-e5	
2021-02-01	2023-01-19 08:51	2023-01-19 08:51		e139927, 139927	
2021-01-06	2023-01-19 08:51	2023-01-19 08:51		103981	
2021-06-08	2023-01-19 08:51	2023-01-19 08:51		e147464, 147464	
2021-08-02	2023-01-19 08:51	2023-01-19 08:51		3747-3759	
2021-12	2023-01-19 08:50	2023-01-19 08:50		1819-1832	
2021-09-27	2023-01-19 08:50	2023-01-19 08:50		1882-1892	
2021-06	2023-01-19 08:50	2023-01-19 08:50		821-828	
2021-02	2023-01-19 08:50	2023-01-19 08:50		244-254	
2021-01-25	2023-01-19 08:50	2023-01-19 08:50		295-305	
2020-02-01	2023-01-19 08:50	2023-01-19 08:50		291-297	
2020-01-27	2023-01-19 08:49	2023-01-19 08:49		1242	
2020-06-14	2023-01-19 08:49	2023-01-19 08:49		4236	
2020-01	2023-01-19 08:49	2023-01-19 08:49		38-47	
2020-03	2023-01-19 08:48	2023-01-19 08:48		435-440	
2020-10	2023-01-19 08:48	2023-01-19 08:48		107665	
2019-08-10	2023-01-19 08:48	2023-01-19 08:48		488-496	
2019	2023-01-19 08:47	2023-01-19 08:47		5063089	
2019-09	2023-01-19 08:47	2023-01-19 08:47		675-681	
2019-09	2023-01-19 08:44	2023-01-19 08:44		1760-1768	
2018-11	2023-01-18 21:34	2023-01-18 21:34		2722-2733	
2018	2023-01-18 21:34	2023-01-18 21:34		7659243	
2018-09-21	2023-01-18 21:34	2023-01-18 21:34		246	
2018-11-26	2023-01-18 21:33	2023-01-18 21:33		483	
2018-10	2023-01-18 21:33	2023-01-18 21:33		1731-1739	
2018-03-01	2023-01-18 21:32	2023-01-18 21:32		441-449	

2018	2023-01-18 21:32	2023-01-18 21:32		389-405
2017-11	2023-01-18 21:32	2023-01-18 21:32		3373-3382
2017-12	2023-01-18 21:32	2023-01-18 21:32		2351-2360
2017-12-11	2023-01-18 21:32	2023-01-18 21:32		17328
2021-04-26	2023-01-18 21:32	2023-01-18 21:32		811-818
2017-12-01	2023-01-18 21:31	2023-01-18 21:31		2079-2089
2017-11	2023-01-18 21:31	2023-01-18 21:31		1066-1075
2022	2023-01-18 21:31	2023-01-18 21:31		e0262667
2016-01-21	2023-03-06 10:41	2023-03-06 10:41		e84916
2022	2023-03-07 09:43	2023-03-07 09:43	2023-03-07 09:43	372-387
2008-09	2023-03-12 14:05	2023-03-12 14:05	2023-03-12 14:05	595-605
2019-04-12	2023-03-14 15:03	2023-03-14 15:03	2023-03-14 15:03	499

Issue	Volume	Number Of Volumes	Journal Abbreviation
1	27		Lupus
1	14		Toxins (Basel)
5	12		Proteomics Clin Appl
1	7		Sci Rep
11	32		Nephrol Dial Transplant
1	17		Cardiovasc Diabetol
1	34		Pediatr Nephrol
10	14		PLoS One
1	18		BMC Nephrol
7	33		Pediatr Nephrol
11	5		Kidney Int Rep
18	20		Int J Mol Sci
9	17		Clin J Am Soc Nephrol
3	10		Biomedicines
2	28		J Am Soc Nephrol
1	8		Sci Rep
12	36		Nephrol Dial Transplant
4	8		Lancet Diabetes Endocrinol
3	9		Proteomes
4	9		Proteomes
1	37		Nephrol Dial Transplant
3	32		Nephrol Dial Transplant
2	33		Nephrol Dial Transplant
1	42		Diabetes Care
3	131		J Clin Invest
	230		J Proteomics
11	6		JCI Insight
8	60		Rheumatology (Oxford)
6	34		J Nephrol
10	36		Nephrol Dial Transplant
3	34		J Nephrol
2	73		Arthritis Rheumatol
2	36		Nephrol Dial Transplant
2	35		Nephrol Dial Transplant
1	10		Sci Rep
12	21		Int J Mol Sci
1	57		J Med Genet
2	11		J Diabetes Investig
10	34		J Diabetes Complications
10197	394		Lancet
	2019		J Immunol Res
9	33		J Diabetes Complications
9	42		Diabetes Care
11	29		J Am Soc Nephrol
	2018		Mediators Inflamm
1	19		BMC Nephrol
12	7		J Clin Med
10	33		Pediatr Nephrol
3	33		Nephrol Dial Transplant

2	43	Kidney Blood Press Res
11	28	J Am Soc Nephrol
12	32	Pediatr Nephrol
1	7	Sci Rep
5	36	Nephrol Dial Transplant
12	32	Nephrol Dial Transplant
6	2	Kidney Int Rep
1	17	PLoS One
1	1	JCI Insight
5	53	Am J Nephrol
3	52	American Journal of Kidney Diseases
4	8	JCM
		Kidney Int.

Short Title
Collagen-Derived Peptides in CKD
Association of kidney fibrosis with urinary peptides
Urinary Dickkopf-3
Early detection of diabetic kidney disease by urinary proteomics and subsequent intervention with spi
Association between urinary dickkopf-3, acute kidney injury, and subsequent loss of kidney function ir
Urinary biomarkers of tubular injury to predict renal progression and end stage renal disease in type 2

Urinary Epidermal Growth Factor

Series	Series Number	Series Text	Series Title	Publisher	Place	Language
						eng
						eng
						eng
						eng
						eng
						eng
						eng
						eng
						eng
						eng
						eng
						eng
						eng
						eng
						eng
						eng
						eng
						eng
						eng
						eng
						eng
						eng
						eng
						eng
						eng
						eng
						eng
						eng
						eng
						eng
						eng
						eng
						eng
						eng
						eng
						eng
						eng
						eng
						eng
						eng
						eng
						eng
						eng
						eng
						eng
						eng
						eng
						eng
						eng

	eng
	eng
	eng
	eng
	eng
	eng
	eng
	eng
	eng
	en
	en
	en

Rights	Type	Archive	Archive Location	Library Catalog	Call Number
				PubMed	
				PubMed	
				PubMed	
				PubMed	
				PubMed	
				PubMed	
				PubMed	
				PubMed	
				PubMed	
				PubMed	
				PubMed	
				PubMed	
				PubMed	
				PubMed	
				PubMed	
				PubMed	
				PubMed	
				PubMed	
				PubMed	
				PubMed	
				PubMed	
				PubMed	
				PubMed	
				PubMed	
				PubMed	
				PubMed	
				PubMed	
				PubMed	
				PubMed	
				PubMed	
				PubMed	
				PubMed	
				PubMed	
				PubMed	
				PubMed	
				PubMed	
				PubMed	
				PubMed	
				PubMed	
				PubMed	
				PubMed	
				PubMed	
				PubMed	
				PubMed	
				PubMed	
				PubMed	
				PubMed	
				PubMed	
				PubMed	

	PubMed
	PubMed
	PubMed
	PubMed
	PubMed
	PubMed
	PubMed
	PubMed
	PubMed
	DOI.org (Crossref)
	DOI.org (Crossref)
	DOI.org (Crossref)

Extra	Notes
PMID: 28474961 PMCID: PMC6037307	
PMID: 35050988 PMCID: PMC8781252	
PMID: 29611317	
PMID: 29208969 PMCID: PMC5717105	
PMID: 27507891	
PMID: 29625564 PMCID: PMC5889591	
PMID: 30159624 PMCID: PMC6294330	
PMID: 31613925 PMCID: PMC6793943	
PMID: 28158993 PMCID: PMC5291957	
PMID: 29569191	
PMID: 33163723 PMCID: PMC7609973	
PMID: 31510053 PMCID: PMC6770813	
PMID: 35948365 PMCID: PMC9625093	
PMID: 35327386 PMCID: PMC8945118	
PMID: 27624489 PMCID: PMC5280025	
PMID: 30374033 PMCID: PMC6206033	
PMID: 34145894	
PMID: 32135136	
PMID: 34287333 PMCID: PMC8293473	
PMID: 34941814 PMCID: PMC8709096	
PMID: 33313853 PMCID: PMC8719618	
PMID: 27382111	
PMID: 28064163	
PMID: 30455331	
PMID: 33290282 PMCID: PMC7843225	
PMID: 32927112	
PMID: 33974569 PMCID: PMC8262289	
PMID: 33313921 PMCID: PMC8328509	
PMID: 33847920	
PMID: 33068410 PMCID: PMC8633454	
PMID: 33275197 PMCID: PMC8192364	
PMID: 32892508	
PMID: 31598726	
PMID: 30357416	
PMID: 31988353 PMCID: PMC6985249	
PMID: 32545899 PMCID: PMC7352871	
PMID: 31519711	
PMID: 31483944 PMCID: PMC7078097	
PMID: 32653382	
PMID: 31202596	
PMID: 31637265 PMCID: PMC6766169	
PMID: 31227289	
PMID: 31262950	
PMID: 30279273 PMCID: PMC6218861	
PMID: 30158836 PMCID: PMC6109534	
PMID: 30241508 PMCID: PMC6150979	
PMID: 30486327 PMCID: PMC6306863	
PMID: 29948307 PMCID: PMC6132884	
PMID: 29106632	

PMID: 29539623
PMID: 28698269 PMCID: PMC5661292
PMID: 28755073 PMCID: PMC7441589
PMID: 29229941 PMCID: PMC5725589
PMID: 31837226
PMID: 27984204 PMCID: PMC5837301
PMID: 29130072 PMCID: PMC5669285
PMID: 35045102 PMCID: PMC8769332
PMID: 27699213 PMCID: PMC5033928

File Attachments
C:\Users\loren\Zotero\storage\FCRXU2R8\Pejchinovski et al. - 2018 - Urine peptidomic biomarkers fo
; C:\Users\loren\Zotero\storage\EHTZXUWS\Mavrogeorgis et al. - 2021 - Collagen-Derived Peptides in
C:\Users\loren\Zotero\storage\7DGZRTBU\Magalhães et al. - 2018 - Comparison of Urine and Plasma
; C:\Users\loren\Zotero\storage\E9AP5ARG\Magalhães et al. - 2017 - Association of kidney fibrosis wi
; C:\Users\loren\Zotero\storage\6U7B332L\Lindhardt et al. - 2017 - Urinary proteomics predict onset
; C:\Users\loren\Zotero\storage\KDAZ58YK\Currie et al. - 2018 - Urinary proteomics for prediction of
C:\Users\loren\Zotero\storage\9UU43DDS\Brunner et al. - 2019 - Urine biomarkers of chronic kidney
; C:\Users\loren\Zotero\storage\9UN74XRL\Takei et al. - 2019 - Urinary Activin A is a novel biomarker
; C:\Users\loren\Zotero\storage\MFNVYNVZ\Pérez et al. - 2017 - Comparative differential proteomic
; C:\Users\loren\Zotero\storage\9IYN58IM\Gonzalez Guerrico et al. - 2020 - Urinary CD80 Discriminat
; C:\Users\loren\Zotero\storage\MDI857DE\Yang et al. - 2019 - Noninvasive Urinary Monitoring of Pro
; C:\Users\loren\Zotero\storage\MPD4YWSQ\Ou et al. - 2022 - Urinary Galectin-3 as a Novel Biomark
; C:\Users\loren\Zotero\storage\V22TBVNR\Zhou et al. - 2017 - Matrix Metalloproteinase-7 Is a Urina
; C:\Users\loren\Zotero\storage\QVI3NLZ4\Rodríguez-Ortiz et al. - 2018 - Novel Urinary Biomarkers F
; C:\Users\loren\Zotero\storage\GD5SMUV6\Sánchez-Álamo et al. - 2021 - Urinary Dickkopf-3 a new
; C:\Users\loren\Zotero\storage\7GWVJZVA\Tofte et al. - 2020 - Early detection of diabetic kidney dis
; C:\Users\loren\Zotero\storage\FL67JQX6\Catanese et al. - 2021 - A Novel Urinary Proteomics Classifi
; C:\Users\loren\Zotero\storage\RKTYZFK8\Wendt et al. - 2021 - Molecular Mapping of Urinary Compl
; C:\Users\loren\Zotero\storage\TA4PEC56\Rudnicki et al. - 2021 - Urine proteomics for prediction of
; C:\Users\loren\Zotero\storage\RFKSLBXE\Pejchinovski et al. - 2017 - Urine peptidome analysis predi
; C:\Users\loren\Zotero\storage\IT9FP2YG\Lindhardt et al. - 2018 - Predicting albuminuria response t
; C:\Users\loren\Zotero\storage\KBKJG8PG\Zürbig et al. - 2019 - CKD273 Enables Efficient Prediction
; C:\Users\loren\Zotero\storage\9Y5S7WTL\Puthumana et al. - 2021 - Biomarkers of inflammation an
; C:\Users\loren\Zotero\storage\HQSQ4GC5\Menez et al. - 2021 - Urinary EGF and MCP-1 and risk of
; C:\Users\loren\Zotero\storage\AY4JW82R\Davies et al. - 2021 - A panel of urinary proteins predicts
; C:\Users\loren\Zotero\storage\N3BM6Y47\Norvik et al. - 2021 - Urinary excretion of epidermal grow
; C:\Users\loren\Zotero\storage\DMTTERNL\Seibert et al. - 2021 - Dickkopf-3 in the prediction of cont
; C:\Users\loren\Zotero\storage\ACR8SGRB\Lee et al. - 2021 - Urinary chemokine C-X-C motif ligand 1
; C:\Users\loren\Zotero\storage\3JGPP34D\Brondani et al. - 2020 - Urinary peptidomics and bioinform
; C:\Users\loren\Zotero\storage\U6UPKFSQ\Ahn et al. - 2020 - Differential Urinary Proteome Analysis
C:\Users\loren\Zotero\storage\65IE4C8B\Doykov et al. - 2020 - Rapid, proteomic urine assay for moni
; C:\Users\loren\Zotero\storage\7TLBP6HI\Gohda et al. - 2020 - Circulating kidney injury molecule-1 a
; C:\Users\loren\Zotero\storage\9W8VHI3D\Li et al. - 2019 - Urinary NGAL and RBP Are Biomarkers of
; C:\Users\loren\Zotero\storage\VL7AHC5E\Zewinger et al. - 2018 - Dickkopf-3 (DKK3) in Urine Identifi
; C:\Users\loren\Zotero\storage\IQHUB4SK\Żyłka et al. - 2018 - Markers of Glomerular and Tubular Da
; C:\Users\loren\Zotero\storage\SMVUM9EU\Satirapoj et al. - 2018 - Urinary epidermal growth factor
; C:\Users\loren\Zotero\storage\LWXDZ32J\Liao et al. - 2018 - Urinary Proteomics for the Early Diagno
; C:\Users\loren\Zotero\storage\74ML2DG3\Li et al. - 2018 - Urinary epidermal growth factor as a pro
; C:\Users\loren\Zotero\storage\6EWKQXGU\Critselis et al. - 2018 - Cost-effectiveness of screening ty

; C:\Users\loren\Zotero\storage\CA9FISPD\Caterino et al. - 2018 - Urine Proteomics Revealed a Signifi
C:\Users\loren\Zotero\storage\2Y42MJ9Y\Dong et al. - 2017 - Urinary biomarkers of cell cycle arrest a
; C:\Users\loren\Zotero\storage\SDWC4HFJ\Rasmussen et al. - 2017 - Urinary endotrophin predicts di
; C:\Users\loren\Zotero\storage\YRTEEG2N\Verbeke et al. - 2021 - The urinary proteomics classifier ch
; C:\Users\loren\Zotero\storage\ZL56FFKA\Siwy et al. - 2017 - Noninvasive diagnosis of chronic kidney
; C:\Users\loren\Zotero\storage\CZWBUSDP\Pontillo et al. - 2017 - Prediction of Chronic Kidney Diseas
; C:\Users\loren\Zotero\storage\L35QNQHL\Petra et al. - 2022 - Urine peptidome in combination with
; C:\Users\loren\Zotero\storage\STKJ6SZ2\Federico et al. - 2016 - Tubular Dickkopf-3 promotes the de
C:\Users\loren\Zotero\storage\I837EFUB\Cortvrindt et al. - 2022 - Urinary Epidermal Growth Factor A
C:\Users\loren\Zotero\storage\JTRZTNGB\Watson et al. - 2019 - A Novel Multi-Biomarker Assay for No

Link Attachments	Manual Tags
http://www.ncbi.nlm.nih.gov/pubmed/28474961	
http://www.ncbi.nlm.nih.gov/pubmed/35050988	
http://www.ncbi.nlm.nih.gov/pubmed/29611317	
http://www.ncbi.nlm.nih.gov/pubmed/29208969	
http://www.ncbi.nlm.nih.gov/pubmed/27507891	
http://www.ncbi.nlm.nih.gov/pubmed/29625564	
http://www.ncbi.nlm.nih.gov/pubmed/30159624	
http://www.ncbi.nlm.nih.gov/pubmed/31613925	
http://www.ncbi.nlm.nih.gov/pubmed/28158993	
http://www.ncbi.nlm.nih.gov/pubmed/29569191	
http://www.ncbi.nlm.nih.gov/pubmed/33163723	
http://www.ncbi.nlm.nih.gov/pubmed/31510053	
http://www.ncbi.nlm.nih.gov/pubmed/35948365	
http://www.ncbi.nlm.nih.gov/pubmed/35327386	
http://www.ncbi.nlm.nih.gov/pubmed/27624489	
http://www.ncbi.nlm.nih.gov/pubmed/30374033	
http://www.ncbi.nlm.nih.gov/pubmed/34145894	
http://www.ncbi.nlm.nih.gov/pubmed/32135136	
http://www.ncbi.nlm.nih.gov/pubmed/34287333	
http://www.ncbi.nlm.nih.gov/pubmed/34941814	
http://www.ncbi.nlm.nih.gov/pubmed/33313853	
http://www.ncbi.nlm.nih.gov/pubmed/27382111	
http://www.ncbi.nlm.nih.gov/pubmed/28064163	
http://www.ncbi.nlm.nih.gov/pubmed/30455331	
http://www.ncbi.nlm.nih.gov/pubmed/33290282	
http://www.ncbi.nlm.nih.gov/pubmed/32927112	
http://www.ncbi.nlm.nih.gov/pubmed/33974569	
http://www.ncbi.nlm.nih.gov/pubmed/33313921	
http://www.ncbi.nlm.nih.gov/pubmed/33847920	
http://www.ncbi.nlm.nih.gov/pubmed/33068410	
http://www.ncbi.nlm.nih.gov/pubmed/33275197	
http://www.ncbi.nlm.nih.gov/pubmed/32892508	
http://www.ncbi.nlm.nih.gov/pubmed/31598726	
http://www.ncbi.nlm.nih.gov/pubmed/30357416	
http://www.ncbi.nlm.nih.gov/pubmed/31988353	
http://www.ncbi.nlm.nih.gov/pubmed/32545899	
http://www.ncbi.nlm.nih.gov/pubmed/31519711	
http://www.ncbi.nlm.nih.gov/pubmed/31483944	
http://www.ncbi.nlm.nih.gov/pubmed/32653382	
http://www.ncbi.nlm.nih.gov/pubmed/31202596	
http://www.ncbi.nlm.nih.gov/pubmed/31637265	
http://www.ncbi.nlm.nih.gov/pubmed/31227289	
http://www.ncbi.nlm.nih.gov/pubmed/31262950	
http://www.ncbi.nlm.nih.gov/pubmed/30279273	
http://www.ncbi.nlm.nih.gov/pubmed/30158836	
http://www.ncbi.nlm.nih.gov/pubmed/30241508	
http://www.ncbi.nlm.nih.gov/pubmed/30486327	
http://www.ncbi.nlm.nih.gov/pubmed/29948307	
http://www.ncbi.nlm.nih.gov/pubmed/29106632	

http://www.ncbi.nlm.nih.gov/pubmed/29539623
http://www.ncbi.nlm.nih.gov/pubmed/28698269
http://www.ncbi.nlm.nih.gov/pubmed/28755073
http://www.ncbi.nlm.nih.gov/pubmed/29229941
http://www.ncbi.nlm.nih.gov/pubmed/31837226
http://www.ncbi.nlm.nih.gov/pubmed/27984204
http://www.ncbi.nlm.nih.gov/pubmed/29130072
http://www.ncbi.nlm.nih.gov/pubmed/35045102
http://www.ncbi.nlm.nih.gov/pubmed/27699213

Automatic Tags
Biomarkers; Case-Control Studies; Electrophoresis, Capillary; Humans; Lupus Erythematosus, Systemic
Biomarkers; CE-MS; chronic kidney disease; Collagen; collagen alpha-1(I) chain; fibrosis; Fibrosis; Pepti
Chromatography, Liquid; Humans; Kidney; peptide sequencing; Peptides; peptidomics; plasma; Proteo
Adult; Collagen; Electrophoresis, Capillary; Female; Fibrosis; Humans; Kidney; Liquid Biopsy; Male; Mas
Aged; albuminuria; Albuminuria; Angiotensin II Type 1 Receptor Blockers; Benzimidazoles; Biomarkers
Adult; Aged; Albuminuria; Biomarkers; Cross-Sectional Studies; Diabetes; Diabetes Mellitus, Type 2; Di
Adiponectin; Adolescent; Area Under Curve; Biomarker; Biomarkers; Biopsy; Child; Children; Chronicity
Activins; Aged; Anti-Neutrophil Cytoplasmic Antibody-Associated Vasculitis; Biomarkers; Biopsy; Fema
2D-DIGE; Adult; Aged; alpha 1-Antitrypsin; Biomarkers; Calbindin 2; Decision Trees; Electrophoresis, G
B7-1 Antigen; Biomarkers; CD80; Child; Child, Preschool; Children; Chronic kidney disease; Diagnosis, D
CD80; focal segmental glomerulosclerosis; lupus nephritis; minimial change disease; nephrotic syndror
Adrenal Cortex Hormones; Adult; Biomarkers; Creatinine; diagnostics; Disease Progression; Female; Gl
Albumins; Animals; Atrophy; biomarker; Biomarkers; Creatinine; cross-sectional analysis; Fibrosis; glon
galectin-3; kidney disease progression; renal biopsy; renal fibrosis; urinary biomarkers
Animals; beta Catenin; Biomarkers; chronic kidney disease; Fibrosis; Humans; Kidney; Kidney Diseases;
Adult; Aged; Albuminuria; Area Under Curve; Biomarkers; Disease Progression; Early Diagnosis; Female
Biomarkers; chronic kidney disease (CKD) progression; Creatinine; Disease Progression; fibrosis; Glome
Adult; Aged; Albuminuria; Diabetes Mellitus, Type 2; Diabetic Nephropathies; Disease Progression; Ear
biomarkers; fibrosis; IFTA; peptides; urine
biomarker; capillary electrophoresis; CE-MS; complement; kidney disease; peptide; proteomics; urine
Adult; biomarker; Disease Progression; Glomerular Filtration Rate; glomerulonephritis; Glomerulonepl
Adolescent; ADPKD; Adult; Biomarkers; Disease Progression; Electrophoresis, Capillary; ESRD; Female;
Adolescent; Adult; Aged; Albuminuria; diabetes mellitus type II; Diabetes Mellitus, Type 2; Double-Blin
Acute Kidney Injury; Aged; Animals; Biomarkers; Chemokine CCL2; Chitinase-3-Like Protein 1; Chronic
Animals; Humans; Mice; Nephrotic syndrome; Nephrotic Syndrome; Protease activity; Proteases; Prote
Acute Kidney Injury; Aged; Aged, 80 and over; Cardiac Surgical Procedures; Cardiovascular disease; Ch
Adult; Antirheumatic Agents; biomarker; Ceruloplasmin; Chemokine CCL2; Female; Humans; inflamma
Biomarker; Biomarkers; Biopsy; Chronic kidney disease; Diabetic nephropathy; GDF15; Glomerular Filt
chronic kidney disease; clinical epidemiology; Creatinine; Disease Progression; epidermal growth facto
Acute Kidney Injury; Adaptor Proteins, Signal Transducing; Aged; Biomarkers; CI-AKI; Contrast Media; (
Adult; Blotting, Western; Case-Control Studies; Cohort Studies; Cross-Sectional Studies; Disease Progre
Biomarkers; Chemokine CXCL16; CXCL16; Diabetes Mellitus; diabetic kidney disease; Diabetic Nephrop
Biomarkers; Chemokine CCL2; Creatinine; Cross-Sectional Studies; diabetic kidney disease; Diabetic Ne
Aged; Algorithms; Biomarkers; Chromatography, Liquid; Computational Biology; Diabetic Nephropathi
Acid Phosphatase; Adult; Aged; Biomarkers; Calcium-Binding Proteins; Case-Control Studies; Cathepsir
biomarker; Biomarkers; Chromatography, Liquid; Fabry disease; Fabry Disease; Female; Glycolipids; Hu
Aged; Biomarker; Biomarkers; Cross-Sectional Studies; Diabetes Mellitus, Type 2; Diabetic kidney disea
Adult; Aged; Diabetes Mellitus, Type 2; Diabetic kidney disease (DKD); Diabetic Nephropathies; Diagno
Acute Kidney Injury; Adaptor Proteins, Signal Transducing; Aged; Aged, 80 and over; Biomarkers; Cardi
Aged; Albuminuria; Biomarkers; Diabetes Mellitus, Type 2; Diabetic Nephropathies; Female; Humans;
Aged; Albuminuria; Angiotensinogen; Biomarkers; Cohort Studies; Cystatin C; Diabetes Mellitus, Type
Aged; Biomarkers; Collagen Type III; Collagen Type VI; Diabetes Mellitus, Type 1; Diabetic Cardiomyop
Adaptor Proteins, Signal Transducing; Adult; Aged; Aged, 80 and over; Albuminuria; Biomarkers; Chem
Albuminuria; Biomarkers; Creatinine; Cross-Sectional Studies; Cystatin C; Diabetes Mellitus, Type 2; Di
Aged; Albuminuria; Biomarker; Biomarkers; Cardiovascular Diseases; Chemokine CCL2; Creatinine; Dia
early prediction; nephropathy; proteomics
Adolescent; Age Factors; Alport syndrome; Biomarkers; Case-Control Studies; Child; Child, Preschool; (
Albumins; Biomarkers; Cohort Studies; Cost-Benefit Analysis; Diabetes Mellitus, Type 2; Diabetic Neph

Adult; Bardet-Biedl Syndrome; Biomarkers; Case-Control Studies; Female; Fibronectin; Fibronectins; Fi
Acute Kidney Injury; Adolescent; Adult; Biomarker; Cardiac Surgery; Cardiac Surgical Procedures; Child
Acute kidney injury; Acute Kidney Injury; Acute renal failure; Area Under Curve; Biomarkers; Cardiac su
Aged; Biomarkers; Collagen Type VI; Creatinine; Disease Progression; Female; Follow-Up Studies; Glom
Adult; Aged; Albuminuria; Cardiovascular Diseases; cardiovascular risk; chronic kidney disease; CKD273
Adult; Aged; biomarkers; Biomarkers; chronic kidney disease; Female; Humans; Male; Middle Aged; pe
biomarker; chronic kidney disease; clinical science; glomerular filtration rate; peptidomics; proteomics
Aged; Biomarkers; Body Fluids; Case-Control Studies; Databases, Factual; Female; Gene Expression; Ge
Adaptor Proteins, Signal Transducing; Animals; Atrophy; Fibrosis; Intercellular Signaling Peptides and P

Editor	Series Editor	Translator	Contributor	Attorney Agent	Book Author

Cast Member	Commenter	Composer	Cosponsor	Counsel	Interviewer

Producer	Recipient	Reviewed Author	Scriptwriter	Words By	Guest	Number

Edition	Running Time	Scale	Medium	Artwork Size	Filing Date

Application Number	Assignee	Issuing Authority	Country	Meeting Name

Conference Name	Court	References	Reporter	Legal Status	Priority Numbers

Programming Language	Version	System	Code	Code Number	Section	Session

Committee	History	Legislative Body

